# Supplementary material for: COVALENCE STUDY: Immunogenicity and Reactogenicity of a COVID-19 mRNA Vaccine in an Open-Label Cohort of Long-Survivor Patients with Metastatic Lung Cancer
Source: Vaccines (Basel). 2025 Mar 5;13(3):273. doi: 10.3390/vaccines13030273 (PMC11946322; doi:10.3390/vaccines13030273)
Supplement: Supplementary file 1 [file vaccines-13-00273-s001.zip › vaccines-3429179-supplementary.pdf]

SUPPPLEMENTARY MATERIALS

Figure S1 COVALENCE trial design……………………………………………………………………………………….………2

Figure S2 Study design and procedures for exploratory endpoint……………………………………………..………………2

Figure S3 Consort diagram……..……………3

Table S1. Primary vaccination details………………………………………………………………………………………………………4

Table S2. Baseline characteristics……4

Table S3. Outcomes and treatments of post-booster Sars-Cov-2 infection……………………………………………….4

Table S4. Kruskal-Wallis and Dunn's test results in COVALECE cohorts………………………………………………………5

Table S5. Rank correlation coefficient tests for age variable…………………………………………………………………..5

Figure S4. Comparison of anti-S IgG titres in LC patients…………………………………..……………………………..……6

Figure S5. Reactogenicity of BNT-162b2 booster dose in uninfected patients…………………………………..………6

Table S6. *Characteristics of IO-treated included in exploratory cytokine analysis* ……………..…………….…………….7

Table S7. Adverse events in patients with highest cytokine fold change………………………………………..……………8

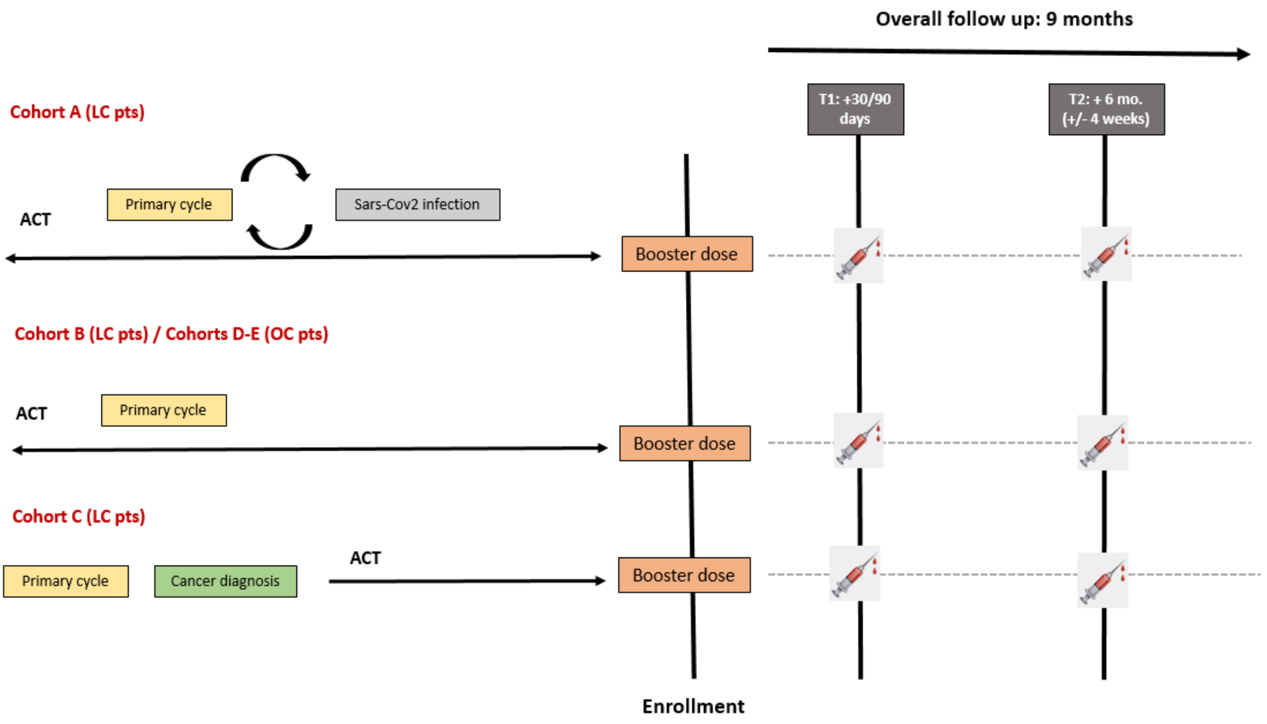

Figure S1 COVALENCE trial design

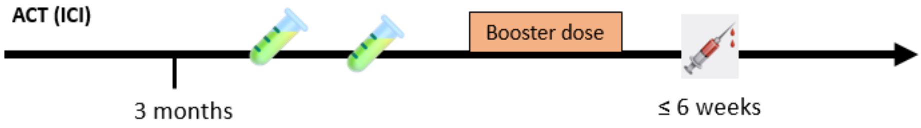

Figure S2 Study design and procedures for exploratory endpoint

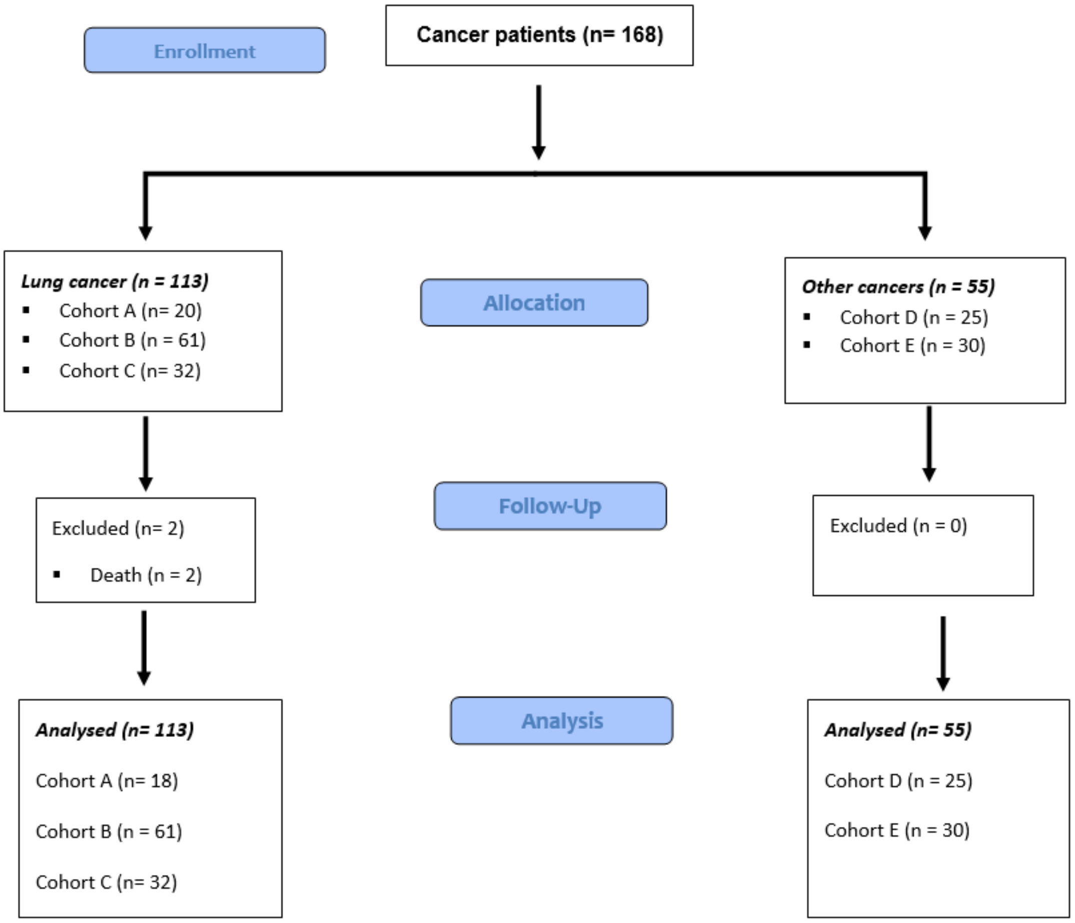

Figure S3 Consort diagram

Table S1. Primary vaccination details

	**Cohort A n = 20**	**Cohort B n = 61**	**Cohort C n = 32**	**Cohort D n = 25**	**Cohort E n = 30**
**Vaccine used**					
Comirnaty (BNT162b2)	15	53	23	25	28
Spikevax (mRNA-1273)	2	3	3	0	1
Vaxzevria (ChAdOx1-S)	2	5	5	0	1
Jcovden (Ad26.COV2.S)	1	0	1	0	0

Table S2. Baseline characteristics

**Cohorts A-B-C-D-E**	**Cohort D-E**
Age: p-value =0.83 (Kruskal Wallis test)<br>Sex: p-value = 0.06 (Fisher test)<br>ECOG: p-value = 0.58 (Fisher test)<br>Line of therapy: p-value=0.02 (Fisher test)	Lung cancer histology:<br>p value=0.22 (Fisher test)

Table S3. Outcomes and treatments of post-booster Sars-Cov-2 infection

	**Cohort A n = 3**	**Cohort B n = 8**	**Cohort C n = 14**	**Cohort D n = 7**	**Cohort E n = 8**
**Outcomes**					
Asymptomatic	1	2	9	0	6
Mild symptomatic	2	6	5	6	2
Hospitalization/UCI	0	0	0	1	0
Death	0	0	0	0	0
**Treatment for Sars-Cov-2 infection**					
None	3	7	9	0	6
Antibiotics	0	0	1	0	2
Antivirals	0	1	1	0	0
Monoclonal antibodies	0	0	3	1	0

Table S4. Kruskal-Wallis and Dunn's test results in COVALECE cohorts

**Kruskal-Wallis test**	***P-value: 0.0019***		
Dunn's test	*Mean rank diff.*	*Significance*	*Adjusted P value*
**Cohort A vs. Cohort B**	**39**	**Yes**	**0.01**
**Cohort A vs. Cohort C**	**42**	**Yes**	**0.02**
Cohort A vs. Cohort D	18	No	>0.99
Cohort A vs. Cohort E	16	No	>0.99
Cohort B vs. Cohort C	2.8	No	>0.99
Cohort B vs. Cohort D	-21	No	0.49
Cohort B vs. Cohort E	-23	No	0.23
Cohort C vs. Cohort D	-24	No	0.47
Cohort C vs. Cohort E	-26	No	0.25
Cohort D vs. Cohort E	-1.8	No	>0.99

Table S5. Rank correlation coefficient tests for age variable

**Spearman's rank correlation coefficient**		
Overall population	Rho -0.30 (95%CI = - 0.44 to - 0.16)*; p (two-tailed) <0.0001*<br>Univariate regression analysis: beta=-0.038, p=0.0009; r2 = 0.07	
Uninfected patients	*Rho -0.21 (95%CI = - 0.38 to - 0.01); p (two-tailed) <0.0001*<br>*Univariate regression analysis: beta=-0.030, p=0.06; r2 = 0.03*	
**Kruskal-Wallis test** P-value: 0.18		
Dunn's test	*Mean rank diff.*	*Significance*
<60 years vs. 60-69 years	5.9	No
<60 years vs. ≥70 years	14	No
Jonckheere-Terpstra test for trends: p=0.07		

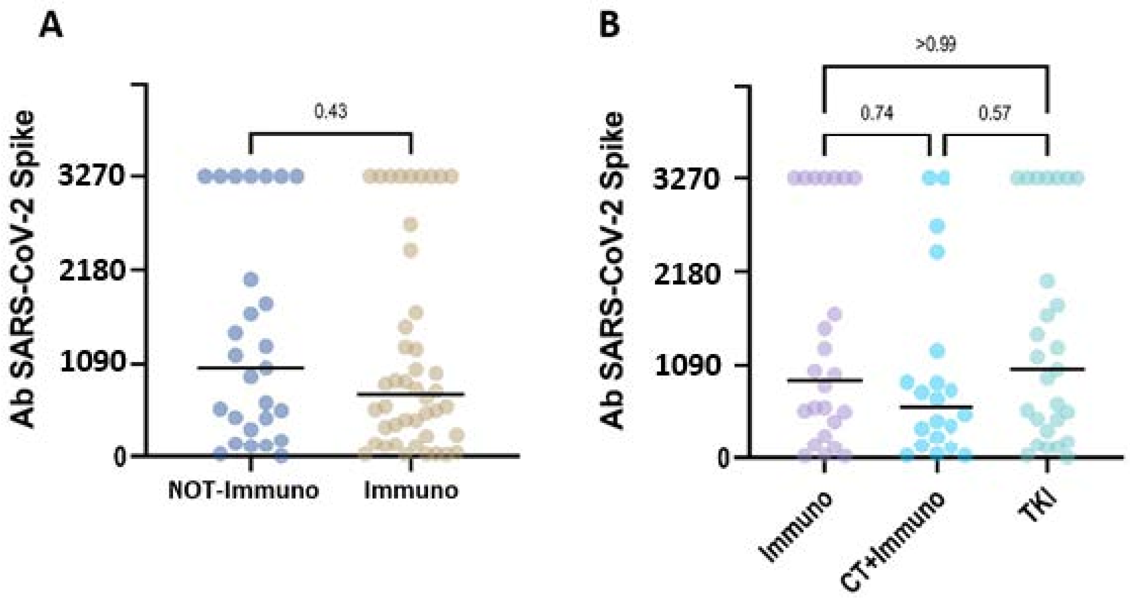

Figure S4 Comparison of anti-S IgG titres in LC patients

In the subgroup of LC patients, no statistically significant difference was observed in IO-treated patients compared to non-IO patients p=0.43) and no difference was also observed when comparing different treatments (IO vs CT+IO vs TKI; p=0.37).

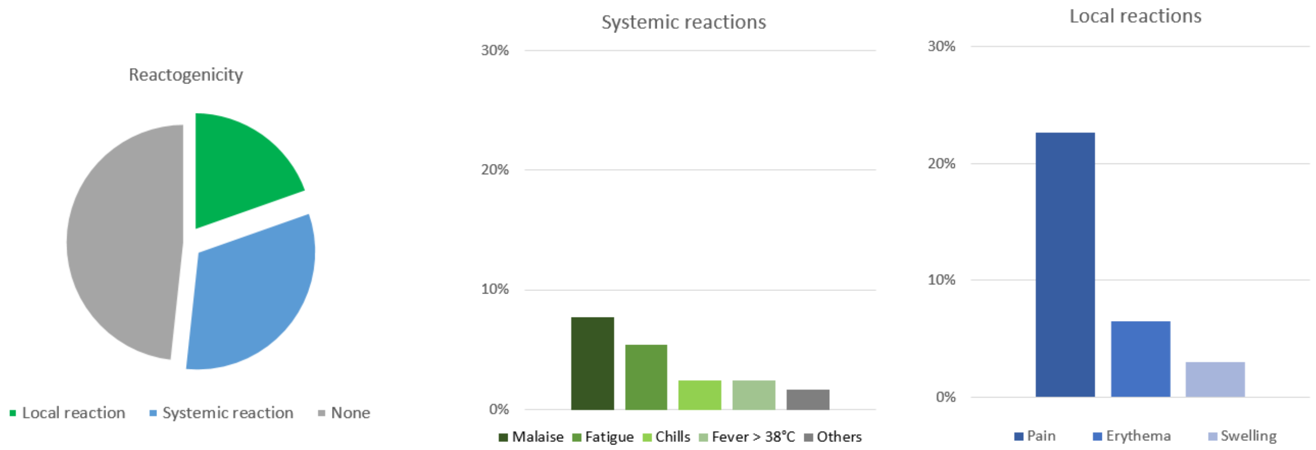

Figure S5. Reactogenicity of BNT-162b2 booster dose in uninfected patients

The majority of participants reported at least one local or systemic symptom after booster dose vaccination. Vaccine reactions have been graded according to CTCAE V.5. Any G3 AE were recorded.

Table S6. Characteristics of IO-treated included in exploratory cytokine analysis

	**Lung cancer patients**	**Other cancer patients**
	**n = 47 (69.1%)**	**n = 21 (30.9%)**
**Age, years**<br>Median (range)	 64 (44 -79)	 65 (34 – 83)
**Sex**<br>Male<br>Female	 30 (63.8%)<br>17 (36.2%)	 14 (66.6%)<br>7 (33.4%)
**Lung cancer histology**<br>Adenocarcinoma<br>Squamous<br>SCLC	 38 (55.9%)<br>3 (4.4 %)<br>6 (8.8 %)	NA
**Other cancers (primary)**<br>Melanoma<br>Renal cell carcinoma<br>HNSCC<br>Merkel cell carcinoma<br>Colorectal cancer	NA	 9 (13.2%)<br>5 (7.3%)<br>3 (4.45%)<br>2 (3%)<br>2 (3%)
**Treatment**<br>CHT + IO<br>Single agent IO<br>Combination IO	 12 (25.5%)<br>33 (70.3%)<br>2 (4.2%)	 -<br>17 (81%)<br>4 (19%)
**Line of therapy**<br>1st<br>≥ 2nd	 37 (75.6%)<br>12 (24.4%)	 14 (66.6%)<br>7 (33.4%)
**Time from Treatment start to BD administration**<br>3-6 months<br>6-12 months<br>≥12 months	 10 (21%)<br>10 (21%)<br>27 (58%)	 2 (9.5%)<br>6 (28.5%)<br>13 (62%)
**List of abbreviations:** CT = chemotherapy; IO = immunotherapy;<br>BD: booster dose		

Table S7. Adverse events in patients with highest cytokine fold change

Patient ID	Alt CKs	Fold change	Diagnosis	IO start	Post-BD sample	AE	CTCA grade	Survival
**Co-C6**	IL-2R<br>IL-6	1.8<br>4.3	Lung adc	3-6 mo.	+ 10 days	None	NA	Alive
**Co-B13**	IL-2R<br>TNF-α	1.5<br>3.4	SCLC	> 12 mo.	+ 18 days	Fatigue	G1	Alive
**Co-B2**	IL-2R<br>IL-6<br>TNF-α	1.5<br>2.2<br>2.0	Lung adc	> 12 mo.	+ 8 days	None	NA	Alive
**Co-B61**	IL-2R<br>TNF-α	1.77<br>2.0	Lung adc	> 12 mo.	+21 days	Fatigue	G1	Alive
**Co-E3**	IL-2R<br>IL-10	1.6<br>2.8	HNSCC	6-12 mo.	+26 days	None	NA	Alive
**Co-E16**	IL-2R<br>IL-10	2.0<br>8.6	Melanoma	> 12 mo.	+ 34 days	Malaise	G1	Alive
**Co-E27**	IL-2R<br>IL-6<br>TNF-α<br>IL-10	3.0<br>6.9<br>1.9<br>2.14	Melanoma	> 12 mo.	+ 24 days	Malaise	G1	Alive
**List of abbreviations: Alt CKs= altered cytokine(s); BD = booster dose; AE = adverse event**<br>**IO start = time from immunotherapy treatment start to BD**								
